# Measuring cell-type specific differential methylation in human brain tissue

**DOI:** 10.1186/gb-2013-14-8-r94

**Published:** 2013-08-30

**Authors:** Carolina M Montaño, Rafael A Irizarry, Walter E Kaufmann, Konrad Talbot, Raquel E Gur, Andrew P Feinberg, Margaret A Taub

**Affiliations:** 1 Medical Scientist Training Program, Johns Hopkins University School of Medicine, 1830 E Monument Street, Baltimore, MD 21205, USA; 2 Predoctoral Training Program in Human Genetics, McKusick-Nathans Institute of Genetic Medicine, Johns Hopkins University School of Medicine, 733 N Broadway, Baltimore, MD 21205, USA; 3 Dana Farber Cancer Institute, Department of Biostatistics and Computational Biology, 450 Brookline Avenue, Boston, MA 02215, USA; 4 Department of Neurology, Boston Children's Hospital and Harvard Medical School, 300 Longwood Avenue, Boston, MA 02115, USA; 5 Department of Psychiatry, University of Pennsylvania, 3400 Spruce Street, Philadelphia, PA 19104, USA; 6 Center for Epigenetics, Johns Hopkins University School of Medicine, 855 N Wolfe Street, Baltimore, MD 21205, USA; 7 Department of Biostatistics, Johns Hopkins Bloomberg School of Public Health, 615 N Wolfe Street, Baltimore, MD 21205, USA

**Keywords:** DNA methylation, epigenetics, differentially methylated region, brain region, cell-type heterogeneity, deconvolution, NeuN, neuron, glia, postmortem brain, fluorescence activated cell sorting

## Abstract

The behavior of epigenetic mechanisms in the brain is obscured by tissue heterogeneity and disease-related histological changes. Not accounting for these confounders leads to biased results. We develop a statistical methodology that estimates and adjusts for celltype composition by decomposing neuronal and non-neuronal differential signal. This method provides a conceptual framework for deconvolving heterogeneous epigenetic data from postmortem brain studies. We apply it to find cell-specific differentially methylated regions between prefrontal cortex and hippocampus. We demonstrate the utility of the method on both Infinium 450k and CHARM data.

## Background

The brain is a particularly good example of highly specialized and diverse functions arising from the same genetic program. Epigenetic mechanisms copy information other than the sequence itself during cell division, such as DNA methylation and chromatin arrangements [1]. Therefore, epigenetics is an attractive substrate for understanding specialized brain function and its disruption in disease. An example of an epigenetic mechanism is DNA methylation, which at CpG dinucleotides is heritable during cell division, because that sequence is recognized by a DNA methyltransferase on newly replicated strands. In post-mitotic cells such as neurons, DNA methylation has been shown to contribute to memory formation [2], other types of synaptic plasticity [3], drug addiction [4], and reversible behavior in the honeybee *Apis mellifera *[5]. Neurological diseases have also been linked to mutations in DNA methyltransferases [6] and methyl-CpG-binding proteins [7].

Despite its importance, the epigenetic profile of the brain has not yet been explored in depth due to, among other factors, brain region and cell-type heterogeneity. The cerebral cortex has distinct functional regions, each organized into cell layers of neurons and glia that vary throughout the cortex [8]. While neurons are the main signaling unit, glia play an important role in scaffolding and maintaining synapses [9]. Epigenetic profiling of neurons and non-neurons using the Illumina GoldenGate assay has shown that neurons and glia have a unique DNA methylation signature that cannot be assessed using samples from bulk cortex [10]. This is important because shifts in glial cell populations such as oligodendrocytes contribute to defects in cortical myelination, and microglia activation has been linked to neurodegenerative disorders [11].

Traditional epidemiological studies using brain tissue done so far do not account for differences in cell-type composition [12-14]. Statistical methods for estimating cell-type composition from genomic profiles have been developed for gene expression [15-18], and DNA methylation in blood tissue [19] and in brain [20]. DNA methylation can then be used to calculate and potentially adjust for differing cell proportions, a crucial step when studying diseases where cell population shifts occur [21].

While DNA methylation data can now be used to calculate differing cell proportions, individual cell-type profiling has not been done yet due to the extensive mixture combinations required for validation in blood (at least five different cell types) [19]. In contrast, cell profiling in the brain can be achieved by separating the cell types into two main compartments: neurons and glia. In a recent publication [20], a method is proposed for estimating neuron and glia proportions similar to the approach proposed for whole blood [19]. While this is a useful step toward correcting for cell distribution, this approach does not permit the unbiased estimation of glia- and neuron-specific differences between two sets of samples [20]. Such calculated cell-type specific analysis offers a crucial advantage in studies of the brain, where neurons and glia cannot generally be dissociated. For example, many brain bank specimens contain pulverized material or even paraffin-fixed specimens, for which methods exist to isolate DNA for genome-scale methylation analysis [22]. Flow sorting, as done here to develop this method, generally does not yield sufficient quantities of material for genome-scale analysis, and is also extremely labor intensive and costly.

Here we have developed a novel statistical epigenetics approach that takes advantage of the stability and cell-type specificity of DNA methylation, as well as the fact that the brain is made up of two major cell types, neurons and glia, in order to deconvolve the two main cell components in the brain. Thus, the method allows one to measure DNA methylation, for example, across brain regions, and from those data calculate to a first approximation the difference in DNA methylation that is neuron- or glia-specific. Moreover, once sorted data is available for a given brain region, investigators can use such data to calculate cell proportions on any unsorted sample measured on the same methylation platform without the need to sort themselves. This approach should have broad application to a range of problems in neurodevelopment and disease research.

## Results and discussion

### Estimation of mixture proportions

We measured DNA methylation profiles for dorsolateral prefrontal cortex (DLPFC), hippocampal formation (HF), and superior temporal gyrus (STG) samples dissected from frozen brains of normal individuals using the comprehensive high-throughput arrays for relative methylation (CHARM) technique [23]. We also labeled and separated neuronal nuclei in a subset of samples using a neuron-specific antibody (NeuN) and fluorescence-activated cell sorting (FACS) [24,25]. Neuronal (NeuN+) and non-neuronal (NeuN-) fractions from DLPFC, HF, and STG were collected for downstream processing and methylation analysis with CHARM (Additional File 1, Figure S1).

**Figure 1:**
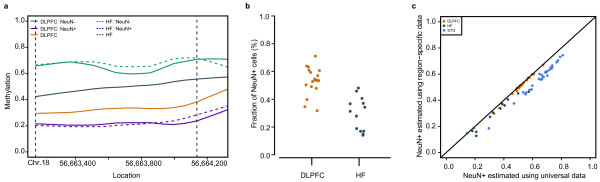
**The proportion of neuronal cells in a given brain region influences the identification of differentially methylated regions**. **(a) **Whole-tissue methylation signals show false-positive brain-region differences. Panel shows a plot of smoothed methylation signals from sorted neuronal and glial cells (teal and purple lines) from DLPFC and HF (solid and dashed lines) as well as from whole-tissue DLPFC (gold line) and HF (grey line). **(b) **Estimated neuronal fraction of cells for whole-tissue samples differs between DLPFC and HF (mean DLPFC = 0.53 (*n *= 19), mean HF = 0.30 (*n *= 13), two-sample t-test *P *value 6.3 × 10^-6^). **(c) **Estimated neuronal fraction of cells for whole-tissue samples using universal DMRs *vs*. estimated neuronal fraction using brain region-specific DMRs from DLPFC (gold), HF (grey), and STG (blue).

To illustrate the downstream effects of the cell population confounding problem, and focusing on two brain regions for clarity, we examined a genomic region for which: (1) no difference was observed between DLPFC and HF in either neuronal or glial fractions; and (2) a difference was observed between neuronal and glial nuclei within each brain region (Figure 1a). Note that a strong methylation difference between brain regions is observed between the non-cell-sorted brain samples. This must be a false-positive and, as we demonstrate below, must be due to differences in cell-type composition between the brain regions.

We modified a statistical method originally developed to estimate cell populations in blood [19] to calculate neuronal and glial proportions for each of our unsorted samples, adapting it to use a constrained linear optimization model (Figure 1b, see overview in Additional File 1, Figure S2a). We confirmed that our approach effectively estimated these cell proportions using a mixture experiment with an independent set of samples (Additional File 1, Figure S2b). To demonstrate that the false-positive results of Figure 1 are due to difference in cell-type distribution, we mathematically reconstructed the unsorted sample methylation profile using the pure neuronal and glial profiles and their estimated frequencies and predicted this result (Additional File 1, Figure S2c).

While the above results rely on having neuronal and glial methylation signals for each brain region, we performed additional analyses to determine whether accurate estimates of neuronal and glial proportions in unsorted samples from a brain region could be obtained using selected data from another brain region. Figure 1c shows the accuracy of estimates obtained from such 'universal' data, compared to estimates based on sorted data from each individual brain region. We also accurately reproduce the cell proportion estimates from our mixture experiment (Additional File 1, Figure S2d, see Materials and Methods for additional details of how this analysis was performed). Our results indicate that accurate estimates could be obtained for a new brain region without the need to sort samples from that region.

### Generative model of methylation signal

Currently, obtaining cell-type specific DMRs from unsorted samples is a mathematically intractable problem. However, because in human postmortem brain samples we are interested in just two cell fractions (neurons and glia), we were able to develop a novel statistical procedure to perform this deconvolution. The methylation signal for any sample *i *at a given genomic location, *Y_i_*, can be modeled as a linear combination of the methylation levels of neuronal and glial fractions in the brain region where the sample *i *was obtained. Specifically, for any given CpG, the DNAm profile of a mixed sample can then be written as (see Materials and Methods):

$$Y_{i}=\mu_{D,+}+\left(\mu_{D,-}-\mu_{D,+}\right)\pi_{i}+\left(\mu_{H,+}-\mu_{D,+}\right)X_{i}\left(1-\pi_{i}\right)+\left(\mu_{H,-}-\mu_{D,-}\right)X_{i}\pi_{i}+\varepsilon_{i}$$

Here, we define $\mu_{D,+}$ and $\mu_{D,-}$ to be the methylation level of neuronal and glial fractions, respectively, in DLPFC, with $\mu_{H,+}$ and $\mu_{H,-}$ defined similarly for HF. For each sample *i*, $X_{i}$ is 1 if sample  i was obtained from HF and 0 for DLPFC samples. We let $\pi_{i}$ to be the fraction of glia in sample  i, so that $1-\pi_{i}$ is the fraction of neurons. Finally, $\varepsilon_{i}$ represents biological variability and measurement error. The statistical insight is that because the term $\pi_{i}$ can be estimated with high precision (Additional File 1, Figure S2c), it can be treated as fixed. With this assumption in place, the equation above is actually a linear model of the form

$$Y_{i}=\beta_{0}+\beta_{1}\pi_{i}+\beta_{2}X_{i}\left(1-\pi_{i}\right)+\beta_{3}X_{i}\pi_{i}+\varepsilon_{i},$$

in which the parameters $\beta_{2}$ and $\beta_{3}$ represent the quantities we are interested in measuring, that is, the differences in neurons and glia, respectively, between brain regions. We refer to this model as M2. Fitting this linear model by least squares and obtaining estimates for millions of genomic locations is computationally feasible. (Fitting the model for 4 million probes took about 5 seconds on our laptop).

**Figure 2:**
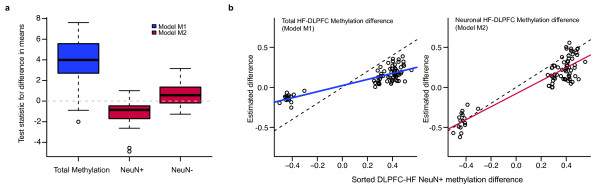
**Effects of direct modeling on false-positives and accuracy**. **(a) **Explicit modeling for differences in cell type reduces false-positive rate. Boxplots of test statistics for the difference in means based on linear regression estimation from models M1 and M2. Eighty percent of regions from M1 show a statistically significant difference in overall mean (at level 0.05); 16% and 12% of regions from M2 show a statistically significant difference in neurons or glia, respectively (at level 0.05). **(b) **Explicit modeling of neuronal methylation differences improves estimation accuracy. Comparison of gold-standard mean difference in methylation in neuron-specific DMRs to the estimated mean difference from models M1 (left) and M2 (right), along with the linear regression fit to the data (95% CI for the slope of the regression line of M1 = (0.29, 0.44), for M2 = (0.68, 0.95).

This statistical framework also exposes the problem with existing naïve approaches to assess DNA methylation signatures in mixed samples. To date, most published analyses ignore cell composition [26-30] and look for associations in a way equivalent to fitting a simple linear regression model $Y_{i}=\alpha_{0}+\alpha_{1}X_{i}+\varepsilon_{i}$ (where the t-test is derived from the $X_{i}=0\;\text{or}\;1$). We refer to this model as M1. In M1, the parameter $\alpha_{1}$ represents a combination of the methylation differences in neurons and glia in which it is impossible to deconvolve cell-type-specific contributions. Furthermore, we can mathematically demonstrate that the least squares estimate of $\alpha_{1}$ will be biased by differences in cell-type frequency under the null hypothesis of no difference in methylation between brain regions (Figure 2a, see Methods Section). Similarly, a naïve model suggested by Guintivano *et al. *[20] that incorporates cell-type proportions $Y_{i}=\gamma_{0}+\gamma_{1}X_{i}+\gamma_{2}\pi_{i}+\varepsilon_{i}$ (we refer to this as model M3) will lead to biased results as well, and to decreased power to detect methylation differences (Additional File 1, Figure S3). We also note that even the superior methods show a small amount of bias (boxplot not centered at 0), which can be explained by slightly inaccurate mixture estimates (see Materials and Methods).

To test the utility of our model, we confirmed our theoretical results with experimental data. First, we obtained estimates of significant neuron-specific methylation differences between DLPFC and HF using sorted brain samples (gold standard, FDR <0.05, Additional File 1, Table S1). We then used the unsorted brain data to calculate the parameters representing the differences in brain-region methylation using models M1 (total methylation difference, $\alpha_{1}$) and M2(neuron-specific methylation difference, $\beta_{2}$). Figure 2b shows that we can estimate neuron-specific methylation differences more accurately with model M2. Therefore, we can assess neuron-specific methylation differences between DLPFC and HF using whole tissue after estimating cell proportions.

Using the sorted samples, we did not find statistically significant DMRs in the non-neuronal fraction, which highlights the importance of isolating a neuronal signal from total methylation values. The result is in agreement with recently published literature suggesting that glia cells, contained in the NeuN- fraction, have less diverse transcription patterns across brain regions than neurons [31], the latter having a distinct DNA-methylation signature [10]. Interestingly, proteins involved in modifying chromatin were found among the brain-region neuronal DMRs, supporting the role of epigenetic mechanisms in neuronal function and synaptic plasticity [32]. For example, neuron-specific methylation of the histone methyltransferase *SETD3*, which methylates histone H3 at lysine 36, was lower in HF than in DLPFC, and histone deacetylase *HDAC4 *shows hypomethylation in DLPFC. Other genes involved in neural differentiation include *JAG1*, *TTL1*, *NPAS4*, *CUX-2*, *DOCK2*, *NGEF*, *OLFM1*, *SATB2*, and *GIT2*.

### Application to Illumina Infinium HumanMethyation450 Dataset

While the CHARM platform has many advantages for studying methylation patterns due to the high density and location of probes, the assay requires restriction-enzyme digestion and lacks single-base resolution. The Illumina Infinium HumanMethylation450 (450K) array has emerged as an affordable alternative to obtain reliable quantitative measurements of methylation. To demonstrate the performance of our method on data from the 450K array, we used data accessible at NCBI GEO database (Guintivano *et al. *[20], accession GSE41826), consisting of 77 normal samples from prefrontal cortex, of which 29 were sorted into neuronal and glial fractions, nine were mixtures of neurons and glia of known proportions, and 10 were unsorted, whole-tissue samples. We first applied our method to obtain accurate cell-fraction estimates on the known mixture samples (Additional File 1, Figure S4a). Using these cell-fraction estimates and the pure neuronal and glial profiles, we mathematically reconstructed the methylation profile for the mixture samples in a set of genomic regions and compared these results to the actual observed methylation for these samples (Additional File 1, Figure S4b). The cell proportion calculations agreed with Guintivano *et al*.'s estimates for prefrontal cortex. Our CHARM cell proportion estimates are on average higher than those obtained using 450K arrays, as the CHARM data were sampled using 2 mm dermal biopsy punches to minimize white matter contamination. The mathematical reconstruction of the methylation signal was also done for the unsorted samples (Additional File 1, Figure S4c).

Given that sorted data on the 450K array are only available for one brain region, we cannot demonstrate our improved ability to detect true brain-region differences in cell-type specific methylation on this platform. However, to show our ability to reduce false-positive signal, we constructed an artificial comparison by grouping the mixture samples with the highest and lowest neuronal fractions and applied models M1 and M2 to look for differences between these two groups. Any such differences are clearly due only to cell-fraction variation, and model M2 reduces the number of false-positive signals (Additional File 1, Figure S4d), as we saw for our CHARM data (Figure 2a). These results indicate that our methods apply well to data from the 450K array.

## Conclusions

We describe an algorithm to address a gap in the analysis of methylation data from complex tissues with varying degrees of cell-type heterogeneity such as the brain. To appropriately measure the methylation differences between two brain cortical regions, we separated a small number of samples of the brain nuclei into neuronal and non-neuronal fractions by cell sorting, and developed a statistical method to account for cell heterogeneity in a set of unsorted samples by decomposing the signal into its two components. Our proposed method takes advantage of the separation of the brain cells into two nuclei fractions. The neuronal fraction encompasses a diverse population of neuronal cells, and the non-neuronal nuclei contain astrocytes, oligodendrocytes, a minority of NeuN-negative neurons, and endothelial cells. To separate the methylation signal into more than two fractions is mathematically plausible, as one can simply define $\pi_{i}$ as the fraction of cells of the cell-type of interest, fit model M2, and consider $\beta_{3}$. However, investigating how robust our results are to the noise in cell fraction estimates when there are more than two cell types will require further study.

The experimental design presented here provides for efficient use of scarce tissue bank resources and limited funds for methylation profiling. Once purified methylation profiles are obtained from the brain regions of interest using a small number of samples, the gold-standard methylation data can be used for any further analysis, and by any laboratory, without the need to sort nuclei again. We have demonstrated our method on data from both CHARM and the Illumina 450K array. To apply our method to a new measurement platform or new brain regions, we recommend performing cell sorting on a subset of the samples to first obtain the cell-type specific signals needed for the cell-fraction estimation. If brain-region specific data are not available, we have also shown that for samples measured with CHARM, accurate estimates of cell proportions in samples from one brain region could be obtained using sorted data from another brain region. We provide a framework that can be applied, even retrospectively, to psychiatric case-control studies using frozen postmortem brain samples, and can be easily adapted to other microarray or sequencing platforms, and to other target tissues.

## Materials and methods

### Generative model of methylation signal

To illustrate our model, we consider the case of estimating differences in methylation between DLPFC (D) and HF (H). We assume these brain tissues are composed of two cell types, NeuN+ (+) and NeuN- (-). For a fixed genomic position, we let *μ*j,k be the methylation level in region *j, j *∈ {*H, D*} and cell type *k, k *∈ {+, -}. Scientifically, we are interested in identifying genomic locations where *μ*H,k - *μ*D,k ≠ 0, that is, where NeuN+ or NeuN- have different methylation levels in the two brain regions.

Given a sample *i *and considering a fixed genomic position, we define *X*_i _as the indicator that sample *i *is from the hippocampus, that is, *X*_i _= 1 if sample *i *is from the hippocampus and 0 otherwise. We also define *π*_i _to be the fraction of sample *i *that consists of NeuN- cells (1 - *π*_i _is the fraction of NeuN+ cells). We can then derive the expected value of the methylation signal of sample *i *at that genomic position as

$$E\left(Y_{i}\right)\;=\;\left\{\pi_{i}\mu_{D,-}+\left(1-\pi_{i}\right)\mu_{D,+}\right\}\left(1\;-\;X_{i}\right)+\left\{\pi_{i}\mu_{H,-}+\left(1-\pi_{i}\right)\mu_{H,+}\right\}\left(X_{i}\right).$$

Rearranging terms gives:

(1) $$E\left(Y_{i}\right)=\mu_{D,+}+\left(\mu_{D,-}-\mu_{D,+}\right)\pi_{i}+\left(\mu_{H,+}-\mu_{D,+}\right)X_{i}\left(1-\pi_{i}\right)+\left(\mu_{H,-}-\mu_{D,-}\right)X_{i}\pi_{i}$$

Suppose we wanted to estimate whether there is a difference in methylation between the two brain regions being considered, H and D. If we fit a model with terms matching those above, that is,

(M2) $$E\left(Y_{i}\right)=\beta_{0}+\beta_{1}\pi_{i}+\beta_{2}X_{i}\left(1-\pi_{i}\right)+\beta_{3}X_{i}\pi_{i}$$

then our estimated coefficients have interpretations equivalent to the generative model in Equation 1. Specifically, we can test the hypothesis of no difference in NeuN+ methylation between D and H (*μ*_H,+ _- *μ*_D,+ _= 0) by testing the hypothesis that *β*_2 _= 0, and the hypothesis of no difference in NeuN- methylation between D and H (*μ*_H,- _- *μ*_D,- _= 0) by testing the hypothesis that *β*_3 _= 0.

From the equations above, we can see that estimating the fraction of cells of each type, *π*_i_, allows us to explicitly find locations with brain-region differences specific to NeuN+ or NeuN- cells.

### Naïve models are biased

In general, *π*_i _is unknown and therefore not included in the linear model, that is, the model

(M1) $$E\left(Y_{i}\right)=\alpha_{0}+\alpha_{1}X_{i}$$

is fitted. However, this model does not account for all the sources of variation in *Y*_i_, and the least squares estimate ${\hat{\alpha }}_{1}$ is a biased estimate of the difference in methylation between H and D under the null hypothesis. To see this, we can write $E\left(\hat{\alpha }\right)={\left(X^{\text{t}}X\right)}^{-1}X^{\text{t}}X\left(Y\right)$, where *X *is the design matrix of the above model and ${\hat{\alpha }}_{1}$ is the vector (${\hat{\alpha }}_{0}$, ${\hat{\alpha }}_{1}$) and the hats represent least squares estimates. For simplicity, we assume equal numbers of samples from H and D. We then have

$$E\left({\hat{\alpha }}_{1}\right)=\mu_{H,+}-\mu_{D,+}+\left(\mu_{H,-}-\mu_{H,+}\right){\bar{\pi }}_{H}-\left(\mu_{D,-}-\mu_{D,+}\right){\bar{\pi }}_{D}$$

Where ${\bar{\pi }}_{\text{j}}$ is the mean fraction of NeuN- cells in region *j*. Under the null hypothesis of no difference between D and H in either + or -, we have $\mu_{\text{H, + }}-\mu_{\text{D, + }}=0$ and also $\left(\mu_{\text{H, - }}-\mu_{\text{H, + }}\right)=\left(\mu_{\text{D, - }}-\mu_{\text{D, + }}\right)=\delta$, which gives

$$E\left({\hat{\alpha }}_{1}\right)=\delta \left({\bar{\pi }}_{\text{H}}-{\bar{\pi }}_{\text{D}}\right).$$

This means that where + and - have different methylation levels (*δ *≠ 0), a difference in the fractions of + and - cells in the different brain regions can lead to false-positive signals of brain region differences in methylation.

Guintivano *et al. *[20] estimate $\pi_{i}$ and propose an *ad hoc *approach to adjust for this that is approximated by fitting the following model

(M3) $$E\left(Y_{i}\right)=\gamma_{0}+\gamma_{1}X_{1}+\gamma_{2}\pi_{i}$$

However, this model does not account for all the sources of variation in *Y*_i _either and the least squares estimate ${\hat{\gamma }}_{1}$ is a biased estimate of the difference in methylation between H and D. To see this, we can write $E\left(\hat{\gamma }\right)={\left(X^{t}X\right)}^{-1}X^{t}E\left(Y\right)$, where *X *is the design matrix of the above model and $\hat{\gamma }$ is the vector $\left({\hat{\gamma }}_{0},{\hat{\gamma }}_{1},{\hat{\gamma }}_{2}\right)$ and the hats represent least squares estimates. For simplicity, we assume equal numbers of samples from H and D. We then have

$$E\left({\hat{\gamma }}_{1}\right)=\mu_{H,+}-\mu_{D,+}+K\left(\left(\mu_{H,-}-\mu_{H,+}\right)-\left(\mu_{D,-}-\mu_{D,+}\right)\right)$$

Where K is a function of the $\pi_{i}$ 's that does not depend on the sample size:

$$K=\frac{{\bar{\pi }}_{H}\left(\frac{1}{2}{\bar{\pi }}_{H}\bar{\pi }+\frac{1}{2}{\bar{\pi }}^{2}-{\left(\bar{\pi }\right)}^{2}\right)+\frac{1}{2}{{\bar{\pi }}^{2}}_{H}\left(\bar{\pi }-{\bar{\pi }}_{H}\right)}{\frac{1}{2}\left({\bar{\pi }}^{2}-{\left({\bar{\pi }}_{H}\right)}^{2}\right)+\bar{\pi }\left({\bar{\pi }}_{H}-\bar{\pi }\right)}$$

With $\bar{\pi }$ and ${\bar{\pi }}_{{}^{H}}$ the average of the $\pi_{i}$ 's in all samples and H samples, respectively, and ${\bar{\pi }}^{2}$ and ${\bar{\pi }}_{H}^{2}$ the average of the $\pi_{i}^{2}$ 's in all samples and H samples, respectively. Note that the bias is directly proportional to the difference between NeuN+ and NeuN- fractions, demonstrating that this approach is incapable of deconvolving these quantities of interest.

### Estimation of mixture proportions

Although we have shown that fitting the mis-specified model, which does not include the cell-fraction terms, can lead to bias under the null hypothesis, the cell fractions for a given sample are unknown *a priori*. At any given methylation site, we are assuming that there is some underlying mean methylation value within each combination of cell type (+, -) and brain region (D, H). If we know these underlying means, we can derive an estimate of the unknown cell fraction at a particular site, given an observed methylation signal and assuming the generative model above. For example, suppose sample *i *is from D and we observe methylation signal *Y*_i _at a given locus. From Equation 1, we have

(2) $$E\left(Y_{i}\right)=\mu_{D,+}+\pi_{i}\left(\mu_{D,-}-\mu_{D,+}\right)=\pi_{i}\mu_{D,-}+\left(1-\pi_{i}\right)\mu_{D,+}$$

If we assume $\mu_{D,+}$ and $\mu_{D,-}$ are known, $\pi_{i}$ is the only unknown in this equation, so it can be estimated. Note that we do need to constrain our estimate of $\pi_{i}$ to be between 0 and 1. Also, the means *μ *are not known, so we collected data to allow us to estimate these means, by measuring methylation in pure cell sorted + or - fractions from each brain region of interest. Given that these methylation measurements have uncertainty, we want to reduce the uncertainty in our estimate of $\pi_{i}$ by using many informative genomic regions. We first select a set of genomic regions where + and - methylation differs. We then find the optimal value of $\pi_{i}$ to explain the observed methylation for sample *i *in these locations, as a function of our estimated means and $\pi_{i}$, subject to the constraint that $\pi_{i}$ is between 0 and 1. This procedure closely follows that presented by Houseman *et al. *[19].

Selection of the genomic locations can be based on a variety of factors, such as the range of observed methylation at these locations, the variance of the methylation estimates, and the length of the region of differential methylation. For our estimation, we chose the 500 genomic regions which were the strongest + *vs*. - DMR candidates in the brain region of interest in relation to the amount of methylation difference and the length of the region showing the methylation difference. We found that our results were quite robust to the number of regions selected, with 500 performing well.

To investigate whether it is absolutely necessary to have sorted data from a given brain region to estimate cell proportions in unsorted data from that region, we identified a set of 'universal' genomic regions. These universal regions had different NeuN+ and NeuN- methylation signals within a brain region, but showed consistent NeuN+ and NeuN- methylation levels across the three brain regions for which we had data (DLPFC, HF, and STG). Many of these + *vs*. - DMR candidates had consistent NeuN+ and NeuN- levels across brain regions, with 14% to 17% of the probes in the + *vs*. - DMRs belonging to genomic regions of consistent signal. We estimated the means *μ *in these regions of consistent signal using sorted data from DLPFC alone, and then performed cell fraction estimation in the unsorted samples from DLPFC, HF, and STG using these mean values. Since we do not know the true cell fractions in these unsorted samples, we used the estimates we had obtained for each brain region using the region-specific DMRs and mean values, as described above, as our gold standard.

All analysis was implemented in R (R Core Team, R: A Language and Environment for Statistical Computing. R Foundation for Statistical Computing: Vienna, Austria, 2012; [33]). The data discussed in this publication have been deposited in NCBI's Gene Expression Omnibus and are accessible through GEO series accession number GSE48610.

### Effect of inaccurate mixture estimates

As previously described, failure to account for differences in cell-mixtures in our samples can lead to biased estimates of brain-region differences under the null hypothesis of no brain region difference. However, inaccurate mixture estimates can also lead to bias. For example, consider the methylation signal in sample *i*

$$E\left(Y_{i}\right)=\beta_{0}+\beta_{1}\pi_{i}+\beta_{2}X_{i}\left(1-\pi_{i}\right)+\beta_{3}X_{i}\pi_{i}$$

Now suppose we have an inaccurate estimate of $\pi_{i}$, called $\pi_{\text{i}}^{*}$, where $\pi_{\text{i}}^{*}=\pi_{\text{i}}+\gamma_{\text{i}}$. Using this inaccurate estimate gives us the following contribution to our regression formulation from sample *i*:

$$\beta_{0}+\beta_{1}{\pi_{i}}^{*}+\beta_{2}X_{i}\left(1-\pi_{i}*\right)+\beta_{3}X_{i}(\pi_{i}*=\beta_{0}+\beta_{1}\left(\pi_{i}+\gamma_{i}\right)+\beta_{2}X_{i}\left(1-\pi_{i}+\gamma_{i}\right)+\beta_{3}X_{i}\left(\pi_{i}+\gamma_{i}\right)$$

$$=\beta_{0}+\beta_{1}\gamma_{i}+\beta_{1}\pi_{i}+\beta_{2}X_{i}+\left(\beta_{3}-\beta_{2}\right)X_{i}\gamma_{i}$$

$$=\beta_{0}+\beta_{1}\gamma_{i}+\beta_{1}\pi_{i}\left(1-\pi_{i}\right)+\beta_{3}X_{i}\pi_{i}+\left(\beta_{3}-\beta_{2}\right)X_{i}\left(\eta_{i}-\pi_{i}\right)-\left(1-\eta_{i}\right)\pi_{i})$$

$$=\beta_{0}+\beta_{1}\gamma_{i}+\beta_{1}\pi_{i}+\beta_{2}X_{i}\left(1-\pi_{i}\right)+\beta_{3}X_{i}\pi_{i}+\left(\beta_{3}-\beta_{2}\right)\eta_{i}X_{i}\left(1-\pi_{i}\right)+\left(\beta_{3}-\beta_{2}\right)\left(1-\eta_{i}\right)X_{i}\pi_{i}$$

$$=\beta_{0}+\beta_{1}\gamma_{i}+\beta_{1}\pi_{i}+\left(\beta_{2}+\left(\beta_{3}-\beta_{2}\right)\eta_{i}\right)X_{i}\left(1-\pi_{i}\right)+\left(\beta_{3}+\left(\beta_{3}-\beta_{2}\right)\left(1-\eta_{i}\right)\right)X_{i}\pi_{i}$$

where *η*_i _is between 0 and 1, and the third line follows from the fact that *γ*_i _must be between $-\pi_{\text{i}}$ and $1-\pi_{\text{i}}$ to ensure that $\pi_{\text{i}}^{*}$ is between 0 and 1. We can see that the coefficient of $X_{\text{i}}\left(1-\pi_{\text{i}}\right)$ is no longer measuring just the quantity we are interested in (the difference between NeuN+ methylation in regions H and D), but it also has an additional factor related to the size of the estimation error, and similarly for the coefficient of $X_{\text{i}}\pi_{\text{i}}$.

### CHARM DNA methylation analysis

Genomic DNA was isolated from brains using the Masterpure kit from Epicentre, according to the manufacturer's protocol. For genome-wide DNA methylation assessment, 1 ug of genomic DNA from each sample was digested, fractionated, labeled, and hybridized to a CHARM array as described [34,35] using a custom Nimblegen 2.1 million feature array assaying 5,114,655 CpG sites. We used the Bioconductor package 'charm' for sample preprocessing along with the package 'bumphunter' for DMR identification and permutation computation.

### Human postmortem brain samples

Fluorescence-activated cell sorting was performed on frozen postmortem dorsolateral prefrontal cortex (*n *= 4), and hippocampal formation (*n *= 4) and superior temporal gyrus (*n *= 3) from individuals not affected with neurological or psychiatric disease. To validate the statistical model, we used nine additional healthy samples from the dorsolateral prefrontal cortex. These samples underwent nuclei extraction and sorting as described below. The model was applied to additional unsorted control samples (19 samples from DLPFC, 13 samples from HF, 31 samples from STG) to deconvolve NeuN+ and NeuN- methylation signatures. All samples were obtained from the bank of the Center for Neurodegenerative Disease Research (CNDR) in the Department of Pathology and Laboratory Medicine at the University of Pennsylvania (directed by Dr John Q Trojanowski, see Additional File 1, Tables S2-4 for demographic information).

### Nuclei extraction, NeuN labeling, and sorting

Total nuclei were extracted via sucrose gradient centrifugation as previously described [25]. A total of 250 mg of frozen tissue per sample was homogenized in 5 mL of lysis buffer (0.32M sucrose, 10 mM Tris pH 8.0, 5 mM CaCl_2_, 3 mM Mg acetate, 1 mM DTT, 0.1 mM EDTA, 0.1% Triton X-100) by douncing 50 times in a 40-mL dounce homogenizer. Lysate was transferred to a 15 mL ultracentrifugation tube and 9 mL of sucrose solution (1.8 M sucrose, 10 mM Tris pH 8.0, 3 mM Mg acetate, 1 mM DTT) was pipetted to the bottom of the tube. The solution was then centrifuged at 27,000 rpm for 2.5 h at 4C (Beckman, L8-80 M; SW28.1 rotor). After centrifugation, the supernatant was removed by aspiration and the nuclei pellet was resuspended in 500 uL of PBS.

The nuclei were incubated in a staining mix (0.71% normal goat serum, 0.036% BSA, 1:1200 anti-NeuN NeuN (Millipore, MAB377), 1:1400 Alexa647 goat anti-mouse secondary antibody (Invitrogen, 21236) for 45 min by rotating in the dark at 4°C. Unstained nuclei and nuclei stained with only secondary antibody served as negative controls. The fluorescent nuclei were run through a FACS machine with proper gate settings. A small portion of the NeuN^+ ^and NeuN^- ^nuclei were re-run on the FACS machine to validate the purity. Immunonegative (NeuN^-^) nuclei were collected in parallel. To pellet the sorted nuclei, 2 mL of sucrose solution, 50 uL of 1 M CaCl_2_, and 30 uL of Mg acetate were added to 10 mL of nuclei in PBS, incubated on ice for 15 min, then centrifuged at 3,000 rpm for 20 min. The nuclei pellet was resuspended in 10 mM Tris (pH 7.5), 4 mM MgCl_2_, and 1 mM CaCl_2_. Fluorescent images were taken on a Zeiss Axio Observer. Z1 microscope with a Plan-Apochromat 100x/1.40 oil-immersion objective lens. Images were generated using an Axiocam MR3 microscope camera and Axiovision software (AxioVs40, version 4.8.2.0, Carl Zeiss, Inc). Images were processed using ImageJ.

## Abbreviations

CHARM: comprehensive high-throughput arrays for relative methylation; DLPFC: dorsolateral prefrontal fortex; DMR: differentially methylated region; FACS: fluorescence-activated cell sorting; FDR: false discovery rate; HF: hippocampal formation; NeuN+: NeuN-positive fraction; NeuN-: NeuN-negative fraction; STG: superior temporal gyrus.

## Competing interests

The authors declare that they have no competing interests.

## Authors' contributions

CMM conceived of the study, designed and performed experiments, analyzed the data and developed the statistical method, and wrote the paper. MAT conceived of the study, analyzed the data and developed the statistical method, and wrote the paper. APF conceived of the study. RAI analyzed data and developed the statistical method. WEK designed experiments. KT selected and acquired samples. REG selected and acquired samples. All authors read and approved the final manuscript.

## Supplementary Material

Additional file 1 **Supplementary Information**. A PDF file containing Figures S1-4 and Tables S1-4. Click here for file

## References

[B1] Feinberg AP Phenotypic plasticity and the epigenetics of human disease Nature 2007 14 433 440 17522677 10.1038/nature05919

[B2] Miller CA Sweatt JD Covalent modification of DNA regulates memory formation Neuron 2007 14 857 869 17359920 10.1016/j.neuron.2007.02.022

[B3] Feng J Zhou Y Campbell SL Le T Li E Sweatt JD Silva AJ Fan G Dnmt1 and Dnmt3a maintain DNA methylation and regulate synaptic function in adult forebrain neurons Nat Neurosci 2010 14 423 430 20228804 10.1038/nn.2514 PMC3060772

[B4] LaPlant Q Vialou V Covington HE Dumitriu D Feng J Warren BL Maze I Dietz DM Watts EL Iniguez SD Koo JW Mouzon E Renthal W Hollis F Wang H Noonan MA Ren Y Eisch AJ Bolanos CA Kabbaj M Xiao G Neve RL Hurd YL Oosting RS Fan G Morrison JH Nestrel EJ Dnmt3a regulates emotional behavior and spine plasticity in the nucleus accumbens Nat Neurosci 2010 14 1137 1143 20729844 10.1038/nn.2619 PMC2928863

[B5] Herb BR Wolschin F Hansen KD Aryee MJ Langmead B Irizarry R Amdam GV Feinberg AP Reversible switching between epigenetic states in honeybee behavioral subcastes Nat Neurosci 2012 14 1371 1373 22983211 10.1038/nn.3218 PMC3518384

[B6] Hansen RS Wijmenga C Luo P Stanek AM Canfield TK Weemaes CM Gartler SM The DNMT3B DNA methyltransferase gene is mutated in the ICF immunodeficiency syndrome Proc Natl Acad Sci USA 1999 14 14412 14417 10588719 10.1073/pnas.96.25.14412 PMC24450

[B7] Amir RE Van den Veyver IB Wan M Tran CQ Francke U Zoghbi HY Rett syndrome is caused by mutations in X-linked MECP2, encoding methyl-CpG-binding protein 2 Nat Genet 1999 14 185 188 10508514 10.1038/13810

[B8] Kandel ER Schwartz JH Jessell TM Principles of neural science 2000 4 New York: McGraw-Hill, Health Professions Division

[B9] Hughes V Microglia: The constant gardeners Nature 2012 14 570 572 22660301 10.1038/485570a

[B10] Iwamoto K Bundo M Ueda J Oldham MC Ukai W Hashimoto E Saito T Geschwind DH Kato T Neurons show distinctive DNA methylation profile and higher interindividual variations compared with non-neurons Genome Res 2011 14 688 696 21467265 10.1101/gr.112755.110 PMC3083085

[B11] Prinz M Priller J Sisodia SS Ransohoff RM Heterogeneity of CNS myeloid cells and their roles in neurodegeneration Nat Neurosci 2011 14 1227 1235 21952260 10.1038/nn.2923

[B12] Grayson DR Jia X Chen Y Sharma RP Mitchell CP Guidotti A Costa E Reelin promoter hypermethylation in schizophrenia Proc Natl Acad Sci USA 2005 14 9341 9346 15961543 10.1073/pnas.0503736102 PMC1166626

[B13] Mill J Tang T Kaminsky Z Khare T Yazdanpanah S Bouchard L Jia P Assadzadeh A Flanagan J Schumacher A Wang SC Petronis A Epigenomic profiling reveals DNA-methylation changes associated with major psychosis Am J Hum Genet 2008 14 696 711 18319075 10.1016/j.ajhg.2008.01.008 PMC2427301

[B14] Sabunciyan S Aryee MJ Irizarry RA Rongione M Webster MJ Kaufman WE Murakami P Lessard A Yolken RH Feinberg AP Potash JB Genome-wide DNA methylation scan in major depressive disorder PLoS One 2012 14 e34451 22511943 10.1371/journal.pone.0034451 PMC3325245

[B15] Shen-Orr SS Tibshirani R Khatri P Bodian DL Staedtler F Perry NM Hastie T Sarwal MM Davis MM Butte AJ Cell type-specific gene expression differences in complex tissues Nat Methods 2010 14 287 289 20208531 10.1038/nmeth.1439 PMC3699332

[B16] Gaujoux R Seoighe C Semi-supervised Nonnegative Matrix Factorization for gene expression deconvolution: a case study Infect Genet Evol 2012 14 913 921 21930246 10.1016/j.meegid.2011.08.014

[B17] Gong T Hartmann N Kohane IS Brinkmann V Staedtler F Letzkus M Bongiovanni S Szustakowski JD Optimal deconvolution of transcriptional profiling data using quadratic programming with application to complex clinical blood samples PLoS One 2011 14 e27156 22110609 10.1371/journal.pone.0027156 PMC3217948

[B18] Kuhn A Thu D Waldvogel HJ Faull RL Luthi-Carter R Population-specific expression analysis (PSEA) reveals molecular changes in diseased brain Nat Methods 2011 14 945 947 21983921 10.1038/nmeth.1710

[B19] Houseman EA Accomando WP Koestler DC Christensen BC Marsit CJ Nelson HH Wiencke JK Kelsey KT DNA methylation arrays as surrogate measures of cell mixture distribution BMC Bioinformatics 2012 14 86 22568884 10.1186/1471-2105-13-86 PMC3532182

[B20] Guintivano J Aryee MJ Kaminsky ZA A cell epigenotype specific model for the correction of brain cellular heterogeneity bias and its application to age, brain region and major depression Epigenetics 2013 14 290 302 23426267 10.4161/epi.23924 PMC3669121

[B21] Liu Y Aryee MJ Padyukov L Fallin MD Hesselberg E Runarsson A Reinius L Acevedo N Taub M Ronninger M Shchetynsky K Scheynius A Kere J Alfredsson L Klareskog L Ekström TJ Feinberg AP Epigenome-wide association data implicate DNA methylation as an intermediary of genetic risk in rheumatoid arthritis Nat Biotechnol 2013 14 142 147 23334450 10.1038/nbt.2487 PMC3598632

[B22] Gu H Bock C Mikkelsen TS Jager N Smith ZD Tomazou E Gnirke A Lander ES Meissner A Genome-scale DNA methylation mapping of clinical samples at single-nucleotide resolution Nat Methods 2010 14 133 136 20062050 10.1038/nmeth.1414 PMC2860480

[B23] Irizarry RA Ladd-Acosta C Carvalho B Wu H Brandenburg SA Jeddeloh JA Wen B Feinberg AP Comprehensive high-throughput arrays for relative methylation (CHARM) Genome Res 2008 14 780 790 18316654 10.1101/gr.7301508 PMC2336799

[B24] Mullen RJ Buck CR Smith AM NeuN, a neuronal specific nuclear protein in vertebrates Development 1992 14 201 211 1483388 10.1242/dev.116.1.201

[B25] Jiang Y Matevossian A Huang HS Straubhaar J Akbarian S Isolation of neuronal chromatin from brain tissue BMC Neurosci 2008 14 42 18442397 10.1186/1471-2202-9-42 PMC2377267

[B26] Ladd-Acosta C Pevsner J Sabunciyan S Yolken RH Webster MJ Dinkins T Callinan PA Fan JB Potash JB Feinberg AP DNA methylation signatures within the human brain Am J Hum Genet 2007 14 1304 1315 17999367 10.1086/524110 PMC2276356

[B27] Gibbs JR van der Brug MP Hernandez DG Traynor BJ Nalls MA Lai SL Arepalli S Dillman A Rafferty IP Troncoso J Johnson R Zielke HR Ferrucci L Longo DL Cookson MR Singleton AB Abundant quantitative trait loci exist for DNA methylation and gene expression in human brain PLoS Genetics 2010 14 e1000952 20485568 10.1371/journal.pgen.1000952 PMC2869317

[B28] Davies MN Volta M Pidsley R Lunnon K Dixit A Lovestone S Coarfa C Harris RA Milosavljevic A Troakes C Al-Sarraj S Dobson R Schalkwyk LC Mill J Functional annotation of the human brain methylome identifies tissue-specific epigenetic variation across brain and blood Genome Biol 2012 14 R43 22703893 10.1186/gb-2012-13-6-r43 PMC3446315

[B29] Pardo LM Rizzu P Francescatto M Vitezic M Leday GG Sanchez JS Khamis A Takahashi H van de Berg WD Medvedeva YA van de Wiel MA Daub CO Carninci P Heutink P Regional differences in gene expression and promoter usage in aged human brains Neurobiol Aging 2013 14 1825 1836 23428183 10.1016/j.neurobiolaging.2013.01.005

[B30] Hannum G Guinney J Zhao L Zhang L Hughes G Sadda S Klotzle B Bibikova M Fan JB Gao Y Deconde R Chen M Rajapakse I Friend S Ideker T Zhang K Genome-wide methylation profiles reveal quantitative views of human aging rates Mol Cell 2013 14 359 367 23177740 10.1016/j.molcel.2012.10.016 PMC3780611

[B31] Ko Y Ament SA Eddy JA Caballero J Earls JC Hood L Price ND Cell type-specific genes show striking and distinct patterns of spatial expression in the mouse brain Proc Natl Acad Sci USA 2013 14 3095 3100 23386717 10.1073/pnas.1222897110 PMC3581870

[B32] Day JJ Sweatt JD Epigenetic mechanisms in cognition Neuron 2011 14 813 829 21658577 10.1016/j.neuron.2011.05.019 PMC3118503

[B33] R. http://www.r-project.org/

[B34] Jaffe AE Murakami P Lee H Leek JT Fallin MD Feinberg AP Irizarry RA Bump hunting to identify differentially methylated regions in epigenetic epidemiology studies Int J Epidemiol 2012 14 200 209 22422453 10.1093/ije/dyr238 PMC3304533

[B35] Irizarry RA Ladd-Acosta C Wen B Wu Z Montano C Onyango P Cui H Gabo K Rongione M Webster M Ji H Potash JB Sabunciyan S Feinberg AP The human colon cancer methylome shows similar hypo- and hypermethylation at conserved tissue-specific CpG island shores Nat Genet 2009 14 178 186 19151715 10.1038/ng.298 PMC2729128

