# Supplementary material for: Measuring cell-type specific differential methylation in human brain tissue
Source: Genome Biol. 2013 Aug 30;14(8):R94. doi: 10.1186/gb-2013-14-8-r94 (PMC4054676; doi:10.1186/gb-2013-14-8-r94)
Supplement: Additional file 1 — Supplementary Information. A PDF file containing Figures S1-4 and Tables S1-4. [file gb-2013-14-8-r94-S1.PDF]

Measuring cell-type specific differential methylation in human brain tissue

**Figure S1**	Neurons and glia have a distinct methylation profiles across the genome.
**Figure S2**	Validation of cell-fraction estimation method using tissue-specific and universal DMRs.
**Figure S3**	Comparison of three models and their effects on false-positives and accuracy.
**Figure S4**	Cross-platform validation of cell-fraction estimation method using Illumina Infinium Human Methylation450 data.
**Table S1**	DMRs identified between the NeuN+ fractions of Hippocampus and Dorsolateral Prefrontal Cortex.
**Table S2**	Demographic information for the 11 subjects whose samples were used for sorting and CHARM.
**Table S3**	Demographic information for the subjects whose samples were used for validation.
**Table S4**	Demographic information for the subjects whose whole-tissue, unsorted samples were used for cell proportion calculations.

Figure S1

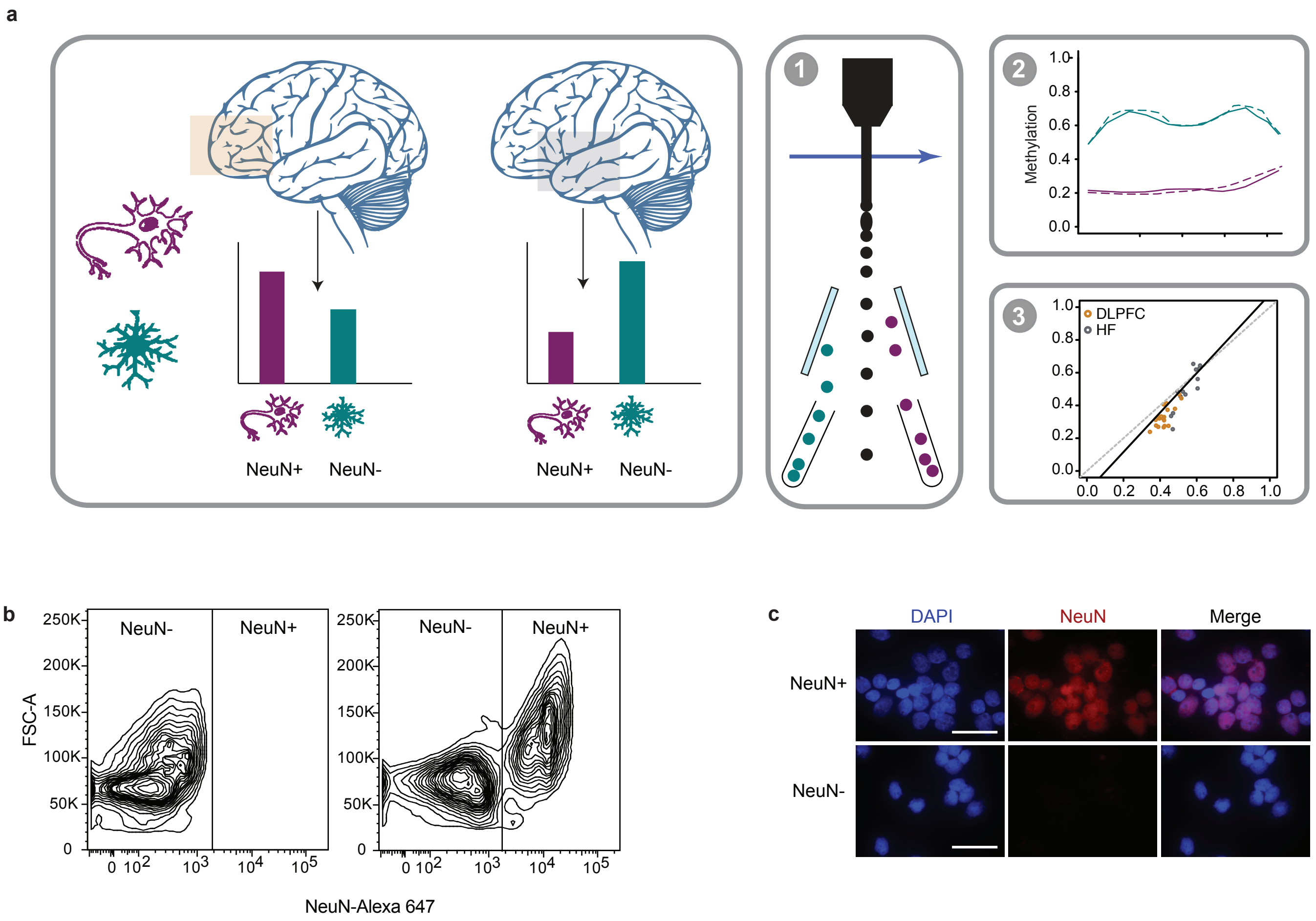

Neurons and glia have a distinct methylation profiles across the genome. (a) Schematic of experimental approach. Steps: 1) separate nuclei via FACS, 2) identify NeuN+ vs. NeuN- DMRs, and 3) compute cell fractions and estimate cell-type specific methylation differences. (b) An example of separation of neuronal and non-neuronal nuclei by FACS. The left panel shows the negative control, and the right panel shows nuclei sorting using Alexa Fluoro 647-conjugated anti-NeuN antibody. (c) Microscopic examination of neuronal (NeuN-Positive, top panel) and non-neuronal nuclei (NeuN-Negative, bottom panel) extracted from prefrontal cortex. Scale bars, 25 μ.

Figure S2

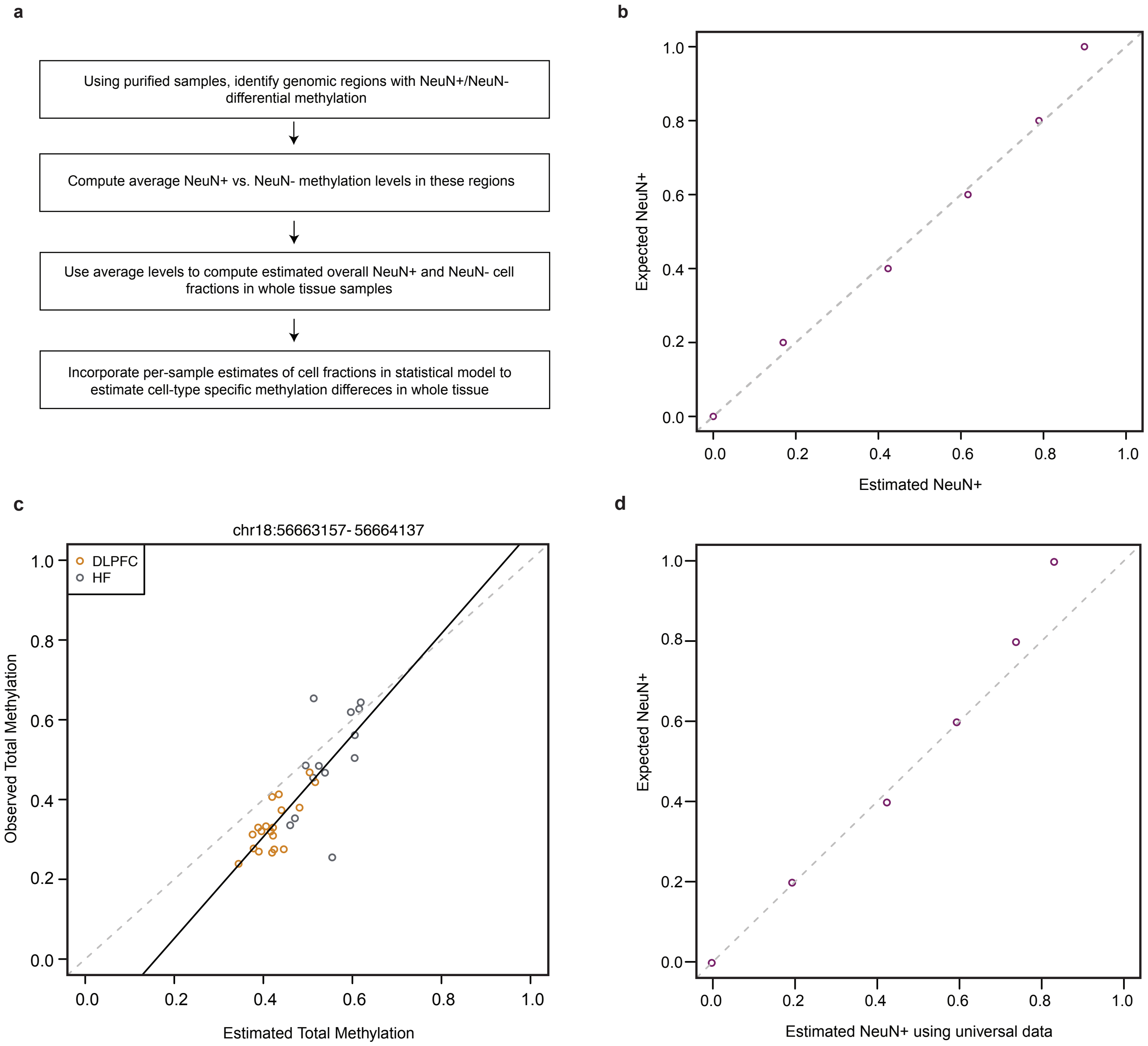

Validation of cell-fraction estimation method using tissue-specific and universal DMRs. (a) Overview of statistical method used to measure proportion of cell types and model cell-type differences between brain regions. (b) Estimated fraction of NeuN+ cells in calibrated data. Expected fraction of NeuN+ cells plotted against estimated, for each of the six known mixture samples (95% CI for the slope of the linear regression = (0.89, 1.22)). (c) Observed vs model-predicted methylation in whole-tissue samples. For the region shown in Fig. 1a, model-predicted mean methylation measures for each DLPFC or HF whole-tissue sample plotted against observed mean methylation measurements (95% CI for the slope = (0.94, 1.61)). (d) Estimated fraction of NeuN+ cells in calibrated data using universal DMRs. Expected fraction of NeuN+ cells plotted against estimated, for each of the six known mixture samples (95% CI for the slope of the linear regression = (0.93, 1.37).

Figure S3

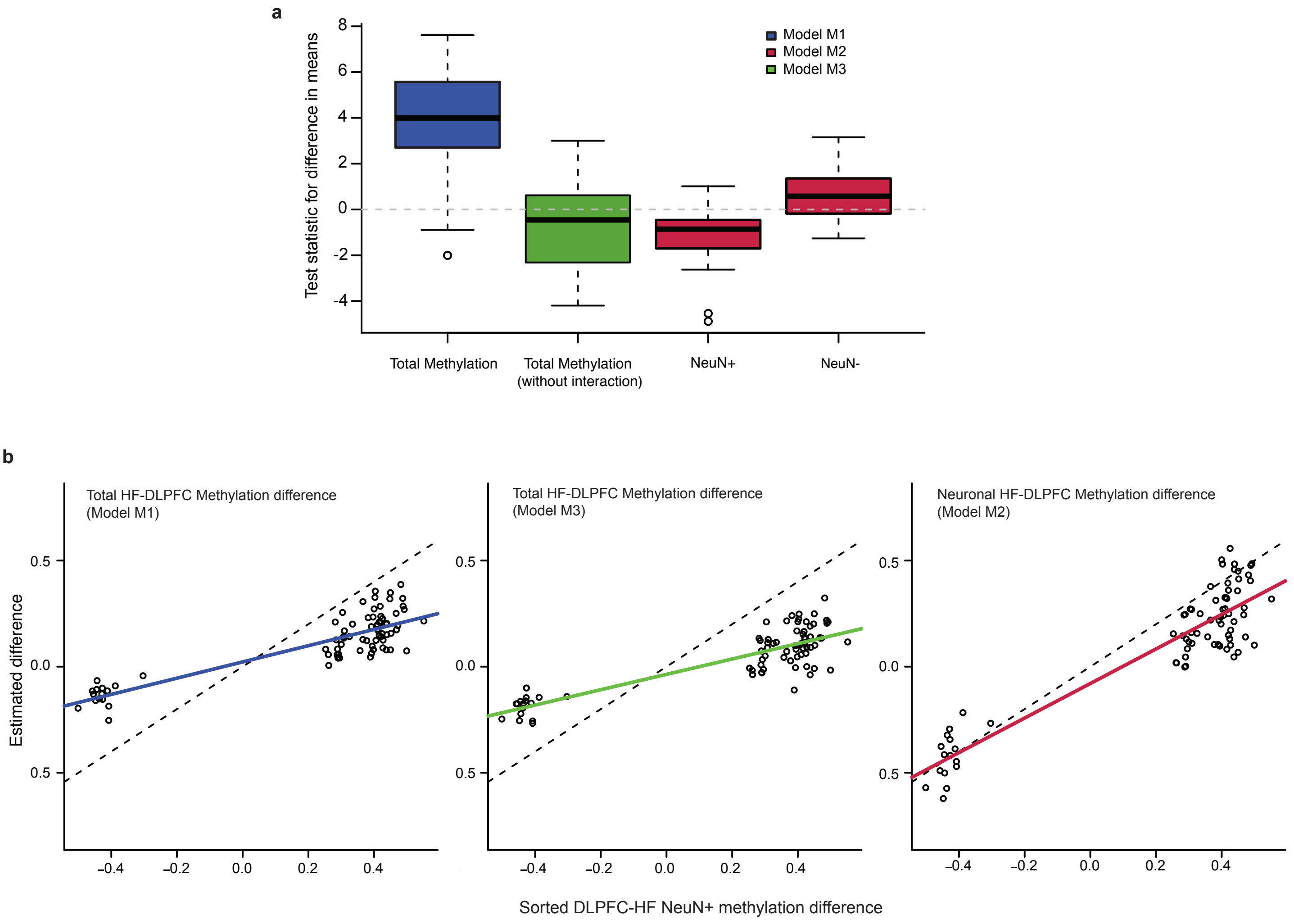

Comparison of three models and their effects on false-positives and accuracy. (a) Explicit modeling for differences in cell type reduces false-positive rate. Boxplots of test statistics for the difference in means based on linear regression estimation from M1, M2, M3. (b) Explicit modeling of NeuN+ methylation differences improves estimation accuracy. Comparison the gold-standard mean difference in methylation in NeuN+ DMRs as measured by our sorted data to the estimated mean difference from models M1 (left), M3 (center), and M2 (right), along with the linear regression fit to the data.

Figure S4

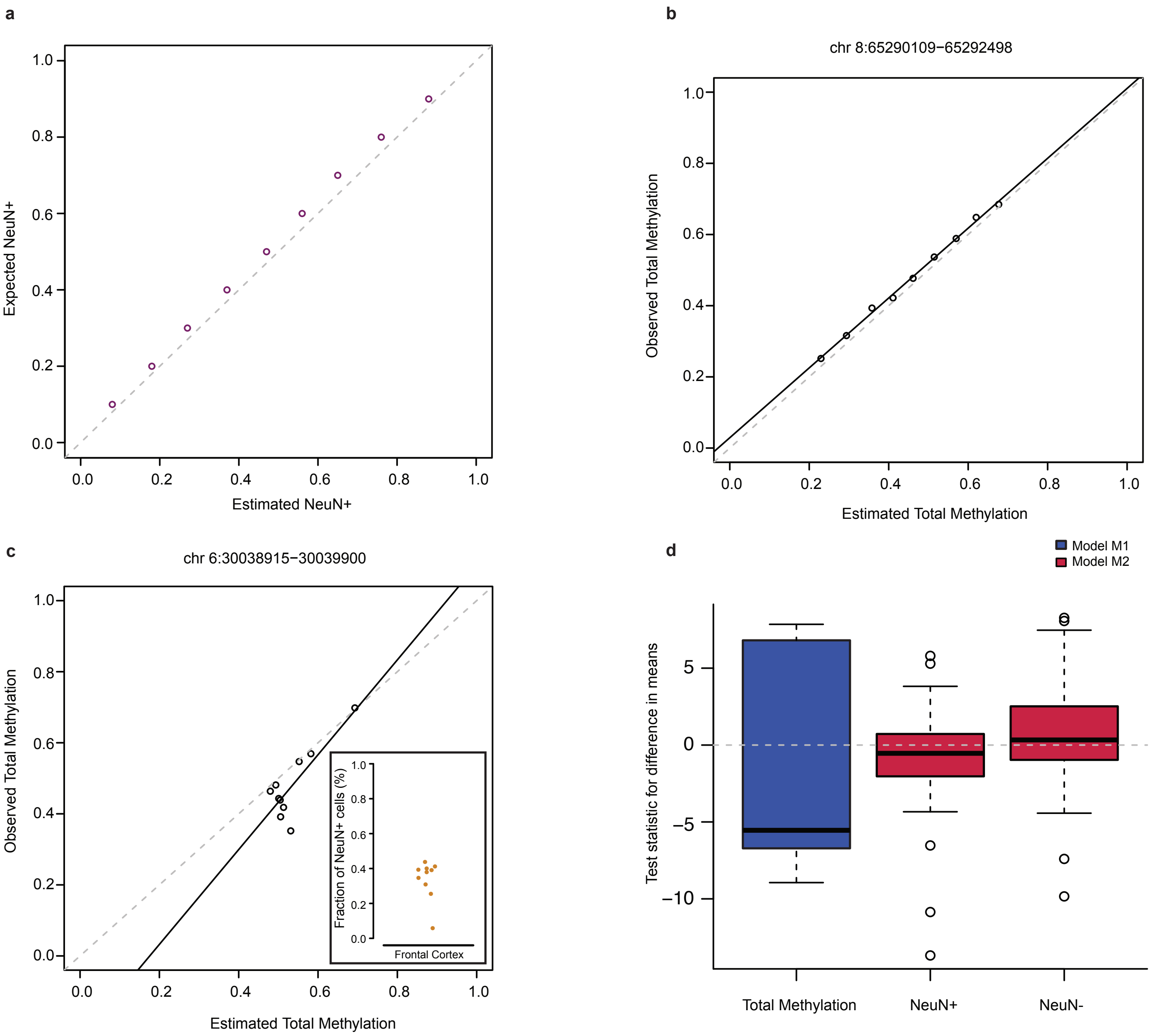

Cross-platform validation of cell-fraction estimation method using Infinium HumanMethylation450 data. (a) Estimated fraction of NeuN+ cells in calibrated data from Guintivano *et al*. Expected fraction of NeuN+ cells plotted against estimated using 450K data, using nine known mixtures (95% CI for the slope of the linear regression = (0.99, 1.05)). (b) Observed vs model-predicted methylation in set of dilution samples. Model-predicted mean methylation measures for each of the nine dilution samples plotted against observed mean methylation measurements (95% CI for the slope = (0.93, 1.03)). (c) Observed vs model-predicted methylation as in (b), using whole tissue. Model-predicted mean methylation measures for each of the whole-tissue samples plotted against observed mean methylation measurements (95% CI for the slope = (0.61, 2.06)). Insert shows the estimated neuronal fraction of cells. (d) Explicit modeling for differences in cell type reduces false-positive rate in 450K data. Boxplots of test statistics for the difference in means based on linear regression estimation from models M1 and M2. 100% of regions from M1 show a statistically significant difference in overall mean (at level 0.05). 12% and 14% of regions from M2 show a statistically significant difference in neurons or glia, respectively (at level 0.05).

Table S1. DMRs identified between the NeuN+ fractions of Hippocampus and Dorsolateral Prefrontal Cortex.

chr	start	end	area	gene	annotation	description	strand	FDR
chr2	37039120	37039610	4.443539234	VIT	NM_053276	inside intron	+	0
chr8	1847802	1850582	11.89279892	ARHGEF10	NM_014629	inside intron	+	0
chr14	99879140	99881527	15.23427008	SETD3	NM_199123	covers exon(s)	-	0
chr3	134444719	134445145	3.256150921	EPHB1	NM_004441	upstream	+	0.01
chr12	110399098	110399580	4.050596254	GIT2	NM_139201	covers exon(s)	-	0.01
chr5	43280159	43280721	4.077190617	MGC42105	NM_153361	inside exon	+	0.01
chr1	88425088	88425642	4.091172379	LMO4	NM_006769	downstream	+	0.01
chr8	11641173	11641729	4.116021713	NEIL2	NM_001135748	inside intron	+	0.01
chr20	10615005	10615632	4.347689131	JAG1	NM_000214	downstream	-	0.01
chr2	2700270	2700914	4.371461694	MYT1L	NM_015025	upstream	-	0.01
chr9	126110153	126110714	4.408873	CRB2	NM_173689	upstream	+	0.01
chr4	152586540	152587166	4.496346065	FAM160A1	NM_001109977	downstream	+	0.01
chr5	79552987	79553619	4.629053385	SERINC5	NM_178276	promoter	-	0.01
chr13	97841718	97842508	4.647641695	MBNL2	NM_144778	upstream	+	0.01
chr19	39165134	39165832	4.764111249	ACTN4	NM_004924	inside intron	+	0.01
chr9	77257015	77257711	4.886381545	RORB	NM_006914	overlaps exon upstream	+	0.01
chr16	15694009	15694630	4.968160497	KIAA0430	NM_014647	covers exon(s)	-	0.01
chr18	72616630	72617539	5.240600888	ZNF407	NM_001146189	inside intron	+	0.01
chr10	3319323	3320093	5.358165546	PITRM1	NM_014889	upstream	-	0.01
chr14	93533158	93533928	5.371861002	ITPK1	NM_014216	inside intron	-	0.01
chr16	14136601	14137391	5.532921973	MKL2	NM_014048	upstream	+	0.01
chr2	233786532	233787304	6.062064385	NGEF	NM_001114090	inside intron	-	0.01
chr22	43481815	43482651	6.295384928	TTLL1	NM_012263	inside intron	-	0.01
chr8	19315281	19316259	6.399131696	CSGALNACT1	NM_001130518	covers exon(s)	-	0.01
chr5	169222175	169223145	6.85926359	DOCK2	NM_004946	inside intron	+	0.01
chr2	200172781	200173879	6.952085002	SATB2	NM_015265	covers exon(s)	-	0.01
chr13	113707930	113709047	7.108499079	MCF2L	NM_024979	inside intron	+	0.01
chr15	52539082	52540210	7.261516407	MYO5C	NM_018728	covers exon(s)	-	0.01
chr1	230333645	230335033	8.008702856	GALNT2	NM_004481	inside intron	+	0.01
chr10	11502874	11504701	8.036172846	USP6NL	NM_001080491	inside exon	-	0.01
chr5	175112809	175114833	8.488052895	HRH2	NM_001131055	overlaps 3'	+	0.01
chr2	240245239	240246533	9.27557277	HDAC4	NM_006037	inside intron	-	0.01
chr2	73234791	73236953	9.391043874	SFXN5	NM_144579	inside intron	-	0.01
chr20	45838118	45840082	9.615809696	ZMYND8	NM_183048	overlaps 3'	-	0.01
chr9	137971767	137974592	10.33741847	OLFM1	NM_006334	inside intron	+	0.01
chr2	101256055	101258440	10.76724963	PDCL3	NM_024065	downstream	+	0.01
chr6	170461840	170464498	10.82805786	LOC154449	NR_002787	downstream	-	0.01
chr8	41573754	41575828	11.15282585	ANK1	NM_020476	covers exon(s)	-	0.01
chr11	66189852	66192238	11.48907956	NPAS4	NM_178864	covers exon(s)	+	0.01
chr20	10649134	10651710	11.8631313	JAG1	NM_000214	inside intron	-	0.01
chr1	13911103	13913166	11.88146905	PDPN	NM_001006624	overlaps 5'	+	0.01
chr8	1308533	1310995	12.72216718	DLGAP2	NM_004745	upstream	+	0.01
chr8	2130797	2133523	12.82866082	MYOM2	NM_003970	downstream	+	0.01
chr4	10086786	10089172	13.47183264	WDR1	NM_005112	inside intron	-	0.01
chr12	111650690	111653035	14.36275222	CUX2	NM_015267	covers exon(s)	+	0.01
chr6	144271110	144271738	3.952381893	PLAGL1	NM_002656	inside intron	-	0.04
chr10	82301270	82301902	4.003159615	SH2D4B	NM_001145719	inside intron	+	0.04
chr3	18435710	18436338	4.135739027	SATB1	NM_002971	overlaps exon downstream	-	0.04
chr21	27194944	27195643	4.371307384	GABPA	NM_002040	downstream	+	0.04
chr15	67700041	67700741	4.392595742	IQCH	NM_001031715	inside intron	+	0.04
chr8	878111	878832	4.522753479	ERICH1	NM_207332	upstream	-	0.04
chr4	54373997	54374770	4.846130189	LNX1	NM_032622	covers exon(s)	-	0.04

chr	start	end	area	gene	annotation	description	strand	FDR
chr18	74938463	74939313	5.129278532	GALR1	NM_001480	upstream	+	0.04
chr12	129283194	129284113	5.558910236	SLC15A4	NM_145648	covers exon(s)	-	0.04
chr18	72843401	72844371	6.184551614	ZADH2	NM_175907	downstream	-	0.04
chr18	74196717	74197701	6.189582852	ZNF516	NM_014643	upstream	-	0.04
chr1	9809315	9810427	6.336957397	CLSTN1	NM_001009566	covers exon(s)	-	0.04
chr14	99858056	99859161	6.484303042	SETD3	NM_032233	downstream	-	0.04
chr2	42565533	42566582	6.566014634	EML4	NM_001145076	downstream	+	0.04
chr2	206551441	206552559	6.862291363	NRP2	NM_201264	inside intron	+	0.04
chr2	97617891	97619103	7.401627122	FAM178B	NM_001122646	inside intron	-	0.04
chr14	91816995	91818823	7.656942855	CCDC88C	NM_001080414	inside intron	-	0.04
chr2	233784409	233785741	7.752771309	NGEF	NM_001114090	covers exon(s)	-	0.04
chr9	98793828	98795228	8.085993671	NCRNA00092	NR_024129	upstream	-	0.04
chr13	21290180	21291528	8.169115031	IL17D	NM_138284	inside intron	+	0.04
chr14	35870200	35872141	8.20357444	NFKBIA	NM_020529	overlaps 3'	-	0.04
chr19	39190539	39192579	8.239046764	ACTN4	NM_004924	covers exon(s)	+	0.04
chr17	80876486	80878706	9.111939115	TBCD	NM_005993	covers exon(s)	+	0.04
chr1	179785533	179787811	9.342791391	FAM163A	NM_173509	downstream	+	0.04
chr6	167171793	167174183	9.955538426	RPS6KA2	NM_001006932	inside intron	-	0.04
chr2	101041834	101042310	3.013924561	CHST10	NM_004854	upstream	-	0.042
chr16	83253486	83253982	3.344500564	CDH13	NM_001257	inside intron	+	0.042
chr5	126924996	126925488	3.448898493	PRRC1	NM_130809	downstream	+	0.042
chr7	70191802	70192292	3.532135383	AUTS2	NM_001127231	inside intron	+	0.042
chr6	126549389	126549883	3.570148657	C6orf173	NM_001012507	upstream	+	0.042
chr19	39189431	39189987	3.935035615	ACTN4	NM_004924	inside intron	+	0.042
chr10	3279310	3282015	9.826597241	PITRM1	NM_014889	upstream	-	0.042
chr19	6268153	6270889	9.95728243	MLLT1	NM_005934	covers exon(s)	-	0.042
chr3	10367730	10370389	10.26507183	ATP2B2	NM_001683	inside exon	-	0.042

Table S2. Demographic information for the 11 subjects whose samples were used for sorting and CHARM

Characteristic	DLPFC[a]	STG[b]	HF[c]
n	4	3	4
Age[d] (years)	84.3(15.2)	82.3(8.3)	76.3(18.8)
Sex (M:F)	2:2	3:0	1:3
PMI[d,e] (hours)	5.7(1.5)	7.0(2.4)	17.1(10.6)
pH[d]	6.4(0.1)	6.5(0.5)	6.4(0.4)

[a]DLPFC= Dorsolateral Prefrontal Cortex
[b]STG = Superior Temporal Gyrus
[c]HF = Hippocampal Formation
[d]Values are given as mean (SD)
[e]PMI = Postmortem Interval

Table S3. Demographic information for the subjects whose samples were used for validation

Sample	Age (years)	Sex	PMI(hours)	pH
DLPFC-1	72	F	4	6.99
DLPFC-3	67	F	5.5	6.16
DLPFC-5	89	F	7	6.3
DLPFC-12	99	F	6	NA
DLPFC-14	89	F	6	6.3
DLPFC-16	88	M	31	6.35
DLPFC-17	76	F	2.9	NA
DLPFC-19	86	F	8	6.78
DLPFC-20	89	M	6.9	6.21
	83.9(10.1)	2:7	8.6(8.5)	6.4(0.3)

Table S4. Demographic information for the subjects whose whole-tissue, unsorted samples were used for cell proportion calculations

**Characteristic**	**DLPFC**[a]	**STG**[b]	**HF**[c]
n	19	31	13
Age[d] (years)	82.9(12.2)	80.4(11.2)	68.1(23.8)
Sex (M:F)	7:12	12:19	4:9
PMI[d,e] (hours)	8.7(6.8)	9.9(6.8)	13(12.9)
pH[d]	6.4(0.3)	6.5(0.3)	6.5(0.3)

[a]DLPFC= Dorsolateral Prefrontal Cortex
[b]STG = Superior Temporal Gyrus
[c]HF = Hippocampal Formation
[d]Values are given as mean (SD)
[e]PMI = Postmortem Interval
